# Hydrocortisone use in France: current practices in 2026

**DOI:** 10.1007/s00431-026-07234-5

**Published:** 2026-07-08

**Authors:** Alexandra Nuytten, Said Maallem, Marie Buttitta, Marine Butin, Cyril Flamant, Olivier Baud

**Affiliations:** 1 Department of Neonatology, Saint Vincent de Paul Hospital, Lille Catholic Hospitals, Lille, France; 2 Delegation for Clinical Research and Innovation, Lille Catholic Hospitals, Lille, France; 3 Department of Neonatology, Hospices Civils de Lyon, Hôpital Femme Mère Enfant, Bron, France; 4 Centre International de Recherche en Infectiologie, Equipe “Pathogénie Des Infections À Staphylocoques”, , INSERM U1111, CNRS UMR 5308, ENS de Lyon, Université Claude Bernard, Lyon 1, Lyon, France; 5 Neonatal Intensive Care Unit, University Hospital of Nantes, Nantes, France; 6 Obstetrical, Perinatal, Paediatric Life Course Epidemiology, OPPaLE, French Institute for Medical Research and Health, Université Paris Cite, CRESS, INSERM, INRAE, Paris, France; 7 Department of Neonatal Medicine and Neonatal Intensive Care, Cochin Port-Royal Hospital, FHU Prem’IMPACT, AP-HP Centre, Paris, France; 8 NeuroDiderot, Université Paris Cité, INSERM, Paris, France

**Keywords:** ELGANs (extremely low gestational age newborn), Postnatal corticosteroids, Hydrocortisone, National survey

## Abstract

Over the past decade, new evidence has emerged regarding postnatal corticosteroids use in extremely preterm infants, particularly early prophylactic hydrocortisone. In France, national recommendations published in 2010 address late postnatal corticosteroids use only and do not incorporate the most evidence-based data. We aimed to provide a national overview of prescribing practices in French neonatal intensive care units (NICUs), focusing on prophylactic hydrocortisone. A national survey was conducted between 1 June and 13 August 2024 among all 66 French level III NICUs using a structured online questionnaire. One response per center was gathered. Fifty-five of 66 (83%) NICUs responded. Prophylactic hydrocortisone was used in 33 (60%) centers: 16 (29%) systematically and 17 (31%) selectively. Among users, 21 (64%) targeted the same population as the PREMILOC study. Non-users cited concerns about benefit–risk balance, lack of evidence, and absence of national guidelines. Fear of neurodevelopmental risk was more frequent among non-users (9/22 (41%) vs 1/33 (3%), *p* < 0.001), while perceived spontaneous intestinal perforation risk did not differ.

*Conclusion*: Hydrocortisone use in French NICUs remain heterogeneous due to safety concerns and a possible uncertainty about the strength of evidence. These findings underscore the need for updated national guidelines to support harmonized, evidence-based care for ELGANs.

**Table:** 

**What is Known:** • *Recent evidence supports prophylactic hydrocortisone to improve survival without bronchopulmonary dysplasia in extremely preterm infants*. • *French national recommendations (2010) address late corticosteroid use (beyond 3 weeks) and do not incorporate recent evidence-based data*.	
**What is New:** • *This national survey provides the first overview of hydrocortisone use in French NICUs, highlighting heterogeneity in prescribing practices*. • *It identifies discrepancies between evidence and clinical practice, supporting the need for updated guidelines*.

**Supplementary Information:**

The online version contains supplementary material available at 10.1007/s00431-026-07234-5.

## Introduction

Postnatal corticosteroids are commonly used in extremely preterm infants to prevent or treat bronchopulmonary dysplasia (BPD), a major cause of morbidity and mortality in this population. However, their use remains controversial due to concerns about potential adverse effects, particularly on neurodevelopmental outcomes [1, 2].

In France, national recommendations published in 2010 mainly address late postnatal corticosteroids use and aim to balance potential respiratory benefits against the risk of long-term complications. These recommendations restrict corticosteroid use to selected high-risk infants, primarily to facilitate extubation or avoid reintubation, and advocate the use of betamethasone after three weeks of life [3]. However, this molecule has not been specifically evaluated in randomized controlled trials for this indication. While its efficacy is widely perceived by clinicians, supporting evidence remains limited, and concerns persist regarding potential adverse effects, particularly on long-term neurodevelopmental outcomes [4, 5].

Since the publication of these guidelines in 2010, new evidence has emerged, particularly regarding early prophylactic hydrocortisone.

The PREMILOC trial demonstrated that early low-dose hydrocortisone improves survival without BPD in extremely preterm infants without increasing the risk of neurodevelopmental impairment [6–8]. These findings have led to growing interest in prophylactic hydrocortisone and have influenced clinical practice in several centers. However, its adoption remains variable, and clinicians may still express concerns regarding its safety and long-term outcomes. This concern may be partly explained by earlier trials evaluating early hydrocortisone, which were prematurely discontinued due to an increased risk of spontaneous intestinal perforation, particularly when combined with indomethacin. Despite extremely recent data from real world cohorts, showing reassuring findings about the safety of early low-dose hydrocortisone [9], a persistent caution remains among clinicians.

Beyond safety concerns, recent data also suggest that the benefit–risk balance of hydrocortisone may depend on patient characteristics. In particular, the context of birth and baseline cortisol levels may influence treatment response, raising questions about the optimal target population for prophylactic hydrocortisone [10]. Accordingly, whether hydrocortisone should be administered systematically or as part of a more targeted approach remains an open question.

This study aims to provide an updated survey of prescribing practices in French neonatal intensive care units, focusing on prophylactic hydrocortisone use, clinicians’ motivations and perceived barriers.

## Methods

### Population

We surveyed all type 3 centers in France (*N* = 66), covering all of establishments caring for the most vulnerable newborns (list of centers in Appendix 1).

### Questionnaire

A structured questionnaire was developed by an expert panel comprising four neonatologists and a methodologist to collect data on current practices for prescribing hydrocortisone (available in supplementary data). It describes:

1. Characterization of the unit: number of annual admissions, status (University hospital of general hospital), specialties available (surgery, sub-specialties, etc.).
2. Prophylactic hydrocortisone practices, administration methods and targeted population, motivations and barriers.

The questionnaire was administered between 1 June and 13 August 2024 via the SPHYNX platform to the heads of department or clinical expert in PNC therapy identified in each center. A second survey round was conducted in November 2025 targeting the 12 non-responding centers in 2024.

### Ethics

Following the French legislation, an approval from ethic committee was not necessary for this study.

### Statistics

A descriptive analysis of the responses was conducted by calculating absolute counts and frequencies for all questions (qualitative variables).

The comparison of motivations and barriers explaining between-group differences for using versus not using prophylactic hydrocortisone was performed using the Chi-square test or Fisher’s exact test in cases of insufficient sample size (according to Cochran’s rule).

A significance threshold of 5% was applied to all analyses. Statistical analyses were conducted using R software (version 4.3.0).

## Results

Of the 66 level 3 centers contacted, 55 (83%) responded to the questionnaire, among them 44 were exclusively dedicated to neonatal intensive care. Regarding PNC prescription practices, 27 (49%) centers reported homogeneous practices within their institution, while 20 (36%) reported relative homogeneity.

The 2010 French guidelines [3] were described as known by 40/55 (73%) of clinicians answering the questionnaire, and partially known by 11/55 (20%). They were declared as strictly applied in 13/55 (24%) centers, often applied in 20/55 (36%), sometimes applied in 10/55 (18%) and never applied in 12/55 (22%). All responders considered that the recommendations needed to be updated.

### Users vs non-users

The use of hydrocortisone for the primary prevention of BPD was reported in 33/55 (60%) centers (“users” opposed to 22 “non users” centers). Among users, 16 centers declared administering hydrocortisone systematically, and 17 selectively based on the ELGANs’ characteristics, and 29/33 (88%) had a written protocol.

Among users centers, 21/33 (64%) applied entry criteria of the PREMILOC trial, while 12/33 (36%) targeted a distinct population, considering factors such as intrauterine growth restriction (IUGR), gestational age, prolonged rupture of membranes, cortisol levels before treatment, the presence of chorioamnionitis, increased oxygen or ventilatory support, and sex.

Among non-users centers, 19/22 (86%) provided main reasons for not prescribing prophylactic hydrocortisone:

- 12/19 (63%) considered the benefit-risk balance to be unfavorable,
- 6/19 (32%) reported insufficient scientific evidence.
- 6/19 (32%) highlighted the lack guidelines published by national societies.

For all centers, the main barriers potentially refraining the prescription of hydrocortisone in ELGANs were:

- the risk of spontaneous intestinal perforation in 18/33 (55%) users and 15/22 (68%) non-users (*p* = 0.48),
- the lack of benefit confirmation from other studies than the PREMILOC trial, mentioned in 14/22 non-users (64%), compared with only 4/33 (12%) of users (*p* = 0.0002).
- the lack of long-term benefits mentioned by 13/22 (59%) of non-users compared with 3/33 (9.1%) of users (*p* = 0.0002).
- a potential neurodevelopmental risk reported by 9/22 (41%) non-users, compared with only 1/33 (3.0%) user (*p* = 0.0005) (Table 1).

**Table 1:** Barriers to the use of early prophylactic hydrocortisone, among all responders, and according to use

Barriers	All responders *N* = 55	Using PHC *N* = 33	Not using PHC *N* = 22	*p*
No barrier	13 (23.6%)	12 (36.4%)	1 (4.5%)	**0.016**
Risk of spontaneous intestinal perforation	33 (60.0%)	18 (54.5%)	15 (68.2%)	0.48
Lack of confirmation of PREMILOC by other studies	18 (32.7%)	4 (12.1%)	14 (63.6%)	**0.0002**
Lack of long-term evidence	16 (29.1%)	3 (9.1%)	13 (59.1%)	**0.0002**
Risk of abnormal neurodevelopmental outcome	10 (18.2%)	1 (3.0%)	9 (40.9%)	**0.0005**
Risk of hyperglycemia	8 (14.5%)	6 (18.2%)	2 (9.1%)	0.45
Risk of infection	7 (12.7%)	4 (12.1%)	3 (13.6%)	1
Risk of HCM	5 (9.1%)	4 (12.1%)	1 (4.5%)	0.64
Risk of hypertension	4 (7.3%)	3 (9.1%)	1 (4.5%)	0.64
Other	2 (3.6%)	1 (3.0%)	1 (4.5%)	0.4

*HCM* hypertrophic cardiomyopathy

### Key factors for using hydrocortisone

The main reason to use prophylactic hydrocortisone, as reported by the 55 responding centers, was the improvement of survival without BPD 32/55 (58%). Other reasons reported were replacement for physiological adrenal insufficiency 15/55 (27%), reduction of invasive ventilation use 12/55 (22%), facilitation of spontaneous closure of the ductus arteriosus 9/55 (16%), beneficial effects on blood pressure 8/55 (15%), and facilitation of extubation 9/55 (16%). These motivations did not significantly differ among prophylactic hydrocortisone users and non-users.

The 33 prescribing centers primarily reported a feeling of improvement in the clinical stability of newborns 12/33 (36%) and a reduction in the need for ductus arteriosus treatment 9/33 (27%). However, 9/33 (27%) of them reported a perceived increase in hyperglycemia requiring insulin therapy.

### Other indications of limitations in hydrocortisone use

Beyond its prophylactic use for BPD prevention, hydrocortisone was also prescribed in 46/55 (84%) for other indications, mainly for hemodynamic support in 45/46 (98%) and substitution in cases of adrenal insufficiency in 27/46 (59%).

Of the 33 centers prescribing hydrocortisone as a preventive measure, 12/33 (36%) systematically measured cortisol levels before starting treatment, 2/33 (6.1%) occasionally did so and 19/33 (58%) did not measure cortisol levels at all.

## Discussion

This national survey provides a comprehensive overview of hydrocortisone use in French NICUs and highlights substantial heterogeneity. Despite emerging evidence supporting its use, adoption remains incomplete, reflecting ongoing uncertainty and safety concerns among clinicians.

The use of prophylactic hydrocortisone varied widely across centers, with only 60% reporting systematic or selective use. The main perceived barriers were concerns about potential adverse effects, particularly spontaneous intestinal perforation and possible long-term neurodevelopmental impairment. Interestingly, perceptions of risk differed between users and non-users: centers not using hydrocortisone reported greater concern regarding neurodevelopmental outcomes, despite available evidence, including the PREMILOC trial, showing no increase in neurodevelopmental impairment and improved survival without BPD [7, 8].

This discrepancy highlights a gap between perceived and evidence-based risks, suggesting that differences in interpretation or dissemination of evidence may influence clinical decision-making. Addressing these perceptions through targeted communication and knowledge translation strategies may therefore represent an important lever to support the implementation of prophylactic hydrocortisone in clinical practice.

Taken together, these results highlight a gap between available evidence and real-world practices that should be carefully considered. While French recommendations from 2010 remain a reference for late PNC use, they do not address prophylactic hydrocortisone and may no longer adequately reflect current evidence or clinical practice.

This study has several limitations. First, the questionnaire was completed by a single respondent from each NICU, usually the head of department. Although responses regarding institutional practices are likely to reflect local protocols, answers to subjective questions, such as perceived barriers to prophylactic hydrocortisone use, may represent the individual views of the respondent rather than those of the entire clinical team. Second, the survey was conducted in two rounds between June 2024 and November 2025, resulting in a prolonged data collection period that may have introduced temporal variability in responses. However, no major evidence or guideline updates regarding prophylactic hydrocortisone use in extremely preterm infants were published during this interval that would be expected to substantially influence clinical practice.

In conclusion, hydrocortisone use in French NICUs appears heterogeneous, due to safety concerns and uncertainty about the strength of evidence.

These findings underscore the need for updated national guidelines to support harmonized, evidence-based care for ELGANs.

## Supplementary Information

Below is the link to the electronic supplementary material.

Supplementary file1 (DOCX 15 KB)

Supplementary file2 (PDF 58 KB)

## Data Availability

The datasets generated and/or analyzed during the current study are not publicly available due to institutional and privacy restrictions but are available from the corresponding author on reasonable request.

## Abbreviations

BPD: Bronchopulmonary dysplasia
ELGANs: Extremely low gestational infants
NICUs: Neonatal intensive care units
PMA: Post menstrual age

## References

[1] Doyle LW, Cheong JL, Hay S, Manley BJ, Halliday HL (2021) Early (<7 days) systemic postnatal corticosteroids for prevention of bronchopulmonary dysplasia in preterm infants. Cochrane Database Syst Rev 2021(10):CD001146. 10.1002/14651858.CD001146.pub6 10.1002/14651858.CD001146.pub6 PMC8530019 34674229

[2] Doyle LW, Cheong JL, Hay S, Manley BJ, Halliday HL (2021) Late (≥7 days) systemic postnatal corticosteroids for prevention of bronchopulmonary dysplasia in preterm infants. Cochrane Database Syst Rev 2021(11):CD001145. 10.1002/14651858.CD001145.pub5 10.1002/14651858.CD001145.pub5 PMC8580679 34758507

[3] Jarreau P-H, Fayon M, Baud O et al (2010) Utilisation de la corticothérapie postnatale chez le nouveau-né prématuré dans la prévention et le traitement de la dysplasie bronchopulmonaire: état des lieux et conduite à tenir. Arch Pediatr 17:1480–1487. 10.1016/j.arcped.2010.07.013 20864322 10.1016/j.arcped.2010.07.013

[4] Remy A, Vincent M, Pastor-Diez B, Picaud J-C (2024) Late postnatal steroid treatment using oral betamethasone can help to close ductus arteriosus in extremely preterm infants who cannot be weaned from ventilation. Eur J Pediatr 184(1):50. 10.1007/s00431-024-05840-9 39604779 10.1007/s00431-024-05840-9 PMC11602834

[5] Löfberg L, Serenius F, Hellstrom-Westas L et al (2025) Postnatal betamethasone treatment in extremely preterm infants and risk of neurodevelopmental impairment: a cohort study. Arch Dis Child Fetal Neonatal Ed 110(4):382–387. 10.1136/archdischild-2024-327360 39694682 10.1136/archdischild-2024-327360 PMC12229056

[6] Baud O, Maury L, Lebail F et al (2016) Effect of early low-dose hydrocortisone on survival without bronchopulmonary dysplasia in extremely preterm infants (PREMILOC): a double-blind, placebo-controlled, multicentre, randomised trial. Lancet 387(10030):1827–1836. 10.1016/S0140-6736(16)00202-6 26916176 10.1016/S0140-6736(16)00202-6

[7] Baud O, Trousson C, Biran V et al (2019) Two-year neurodevelopmental outcomes of extremely preterm infants treated with early hydrocortisone: treatment effect according to gestational age at birth. Arch Dis Child Fetal Neonatal Ed 104(1):F30–F35. 10.1136/archdischild-2017-313756 29321180 10.1136/archdischild-2017-313756

[8] Trousson C, Toumazi A, Bourmaud A et al (2023) Neurocognitive outcomes at age 5 years after prophylactic hydrocortisone in infants born extremely preterm. Dev Med Child Neurol 65(7):926–932. 10.1111/dmcn.15470 36417367 10.1111/dmcn.15470

[9] Smedbäck V, Björklund LJ, Flisberg A et al (2026) Early prophylactic hydrocortisone and bronchopulmonary dysplasia-free survival in extremely preterm infants. JAMA Netw Open 9(2):e2560146. 10.1001/jamanetworkopen.2025.60146 41712209 10.1001/jamanetworkopen.2025.60146 PMC12921520

[10] Renolleau C, Toumazi A, Bourmaud A et al (2021) Association between baseline cortisol serum concentrations and the effect of prophylactic hydrocortisone in extremely preterm infants. J Pediatr 234:65-70.e3. 10.1016/j.jpeds.2020.12.057 33359303 10.1016/j.jpeds.2020.12.057

