# Supplementary material for: Hydrocortisone use in France: current practices in 2026
Source: Eur J Pediatr. 2026 Jul 8;185(8):559. doi: 10.1007/s00431-026-07234-5 (PMC13346289; doi:10.1007/s00431-026-07234-5)
Supplement: Supplementary file 1 — Supplementary file1 (DOCX 15 KB) [file 431_2026_7234_MOESM1_ESM.docx]

Annexe 1

All type 3 centers surveyed in our study.

| Centre Hospitalier | Ville |
| --- | --- |
| CHU AMIENS | AMIENS |
| CHR ANGERS SITE LARREY | ANGERS |
| CH ARGENTEUIL | ARGENTEUIL |
| CH ARRAS | ARRAS |
| CH BASSE TERRE | BASSE TERRE |
| CH DE LA COTE BASQUE - BAYONNE | BAYONNE |
| CHRU JEAN MINJOZ | BESANCON |
| GROUPE HOSPITALIER PELLEGRIN - CHU | BORDEAUX |
| CHRU BREST SITE HOPITAL MORVAN | BREST |
| HOPITAL FEMME MERE ENFANT - HCL | BRON |
| CHU COTE DE NACRE - CAEN | CAEN |
| CH CALAIS | CALAIS |
| CH GUYANNE | CAYENNE |
| CHMS CHAMBERY NH | CHAMBERY |
| CHPC - SITE CHERBOURG | CHERBOURG EN COTENTIN |
| CH ANTOINE BECLERE | CLAMART |
| HOPITAL ESTAING - CHU63 | CLERMONT FERRAND |
| HOPITAL LOUIS MOURIER | COLOMBES |
| CH SUD FRANCILIEN | CORBEILLE ESSONNES |
| CHIC | CRETEIL |
| HOPITAL LE BOCAGE CHRU DIJON | DIJON |
| CHU MARTINIQUE | FORT DE FRANCE |
| CHU GRENOBLE | GRENOBLE |
| CH KEMLIN BICETRE | KREMLIN BICETRE |
| CH Le HAVRE | LE HAVRE |
| CH LENS | LENS |
| HOP JEANNE DE FLANDRE CHU LILLE | LILLE |
| CHU LIMOGE | LIMOGES |
| HOPITAL CROIX-ROUSSE - HCL | LYON 4E ARRONDISSEMENT |
| CENTRE HOSPITALIER DU MANS | MANS |
| APHM HOPITAL NORD | MARSEILLE 15E ARRONDISSEMENT |
| APHM HOPITAL DE LA CONCEPTION | MARSEILLE 5E ARRONDISSEMENT |
| GRAND HOPITAL DE L’EST FRANCILIEN | MEAUX |
| HOPITAL ARNAUD DE VILLENEUVE CHU MPT | MONTPELLIER |
| CH ANDRE GREGOIRE | MONTREUIL |
| HOPITAL EMILE MULLER | MULHOUSE |
| CHRU NANCY - MATERNITE | NANCY |
| CHU DE NANTES SITE HOTEL DIEU HME | NANTES |
| CHU DE NICE HOPITAL DE L'ARCHET | NICE |
| CHU NIMES CAREMEAU | NIMES |
| CHR ORLÉANS - HÔPITAL DE LA SOURCE | ORLEANS |
| HOPITAL NECKER ENFANTS MALADES | PARIS NECKER |
| HOPITAL PORT ROYAL | PARIS PORT ROYAL |
| HOPITAL ROBERT DEBRE | PARIS ROBERT DEBRE |
| HOPITAL TROUSSEAU | PARIS TROUSSEAU |
| CENTRE HOSPITALIER DE PAU | PAU |
| CH PERPIGNAN | PERPIGNAN |
| CHU GUADELOUPE | POINTE A PITRE |
| CHI POISSY | POISSY |
| CHU POITIERS | POITIERS |
| HOPITAL NOVO | PONTOISE |
| HOPITAL MAISON BLANCHE CHU REIMS | REIMS |
| CHRU RENNES SITE HOPITAL SUD | RENNES |
| CHU CHARLES NICOLLE | ROUEN |
| CENTRE HOSPITALIER YVES LE FOLL | SAINT BRIEUC |
| CHU SAINT DENIS Bellepierre | SAINT DENIS – La Réunion |
| CH LA FONTAINE | SAINT DENIS |
| CHU SAINT ETIENNE | SAINT ETIENNE |
| CHU SAINT PIERRE | SAINT – PIERRE – La Réunion |
| CH GHPSO SENLIS | SENLIS |
| HOPITAL DE HAUTEPIERRE | STRASBOURG |
| HOPITAUX MERE & ENFANTS SITE VIGUIER | TOULOUSE |
| CHRU BRETONNEAU - TOURS | TOURS |
| CENTRE HOSPITALIER DE TROYES | TROYES |
| CH VALENCIENNES | VALENCIENNES |
| CHBA SITE DE VANNES | VANNES |
