# Supplementary material for: Hydrocortisone use in France: current practices in 2026
Source: Eur J Pediatr. 2026 Jul 8;185(8):559. doi: 10.1007/s00431-026-07234-5 (PMC13346289; doi:10.1007/s00431-026-07234-5)
Supplement: Supplementary file 2 — Supplementary file2 (PDF 58 KB) [file 431_2026_7234_MOESM2_ESM.pdf]

1. P1_Q1

1) Quel est le nombre approximatif d'admissions d'enfants nés avant 28SA, par an dans votre centre.

<20 ; [20, 40[; [40, 60[; [60, 80[; >80 ; Ne sais pas / Ne se prononce pas

La réponse est obligatoire.

2. P1_Q2

2) Quel est le taux de mortalité chez les enfants nés avant 28SA dans votre centre.

<10% ; [10%, 20%[; [20%, 30%[; [30%, 40%[; >40% ; Ne sais pas / Ne se prononce pas

La réponse est obligatoire.

3. P1_Q3

3) Votre service de réanimation néonatale est:

Une réanimation mixte pédiatrique et néonatale ; Une réanimation néonatale exclusivement ; Autre ; Ne sais pas / Ne se prononce pas

La réponse est obligatoire.

4. P1_Q3_AUTRE

Si "autre" précisez

La réponse est obligatoire.

La question n'est pertinente que si P1_Q3 parmi "Autre".

5. P1_Q4

4) Votre service de réanimation néonatale est un centre (plusieurs choix possible) :

De recours de chirurgie cardiaque ; De recours de chirurgie digestive ; De recours de neurochirurgie ; De recours de chirurgie ophtalmologique ; Hospitalo-universitaire ; Certifié NIDCAP ; Notre centre ne correspond à aucune de ces propositions ; Autre ; Ne sais pas / Ne se prononce pas

La réponse est obligatoire.

Vous pouvez cocher plusieurs cases.

6. P1_Q4_AUTRE

Si "autre" précisez

La réponse est obligatoire.

La question n'est pertinente que si P1_Q4 parmi "Autre".

7. P1_Q5

5) Quel est le taux de Dysplasie Bronchopulmonaire à 36SA (DBP) chez les enfants nés avant 28SA dans votre centre?.

<20% ; [20%, 40%[; [40%, 60%[; [60%, 80%[; >80% ; Ne sais pas / Ne se prononce pas

La réponse est obligatoire.

8. P1_Q6

6) Selon vous, les pratiques de prescription de la corticothérapie postnatale dans votre centre sont.

Homogènes ; Plutôt homogènes ; Plutôt hétérogènes ; Très Hétérogènes ; Ne sais pas / Ne se prononce pas

La réponse est obligatoire.

9. P1_Q7

7) Quel est le nombre d'admissions d'enfants nés avant 32SA, par an en réanimation néonatale dans votre centre.

<50 ; [50 , 100[; [100, 150[; [150, 200[; >200 ; Ne sais pas / Ne se prononce pas

La réponse est obligatoire.

10. P1_Q8

8) Quelle(s) fonction(s) occupez-vous dans le centre? (plusieurs réponses possibles)

Praticien hospitalier ; PUPH ; CCA/Assistant ; Responsable d'unité ; Autre ; Ne sais pas / Ne se prononce pas

La réponse est obligatoire.

Vous pouvez cocher plusieurs cases.

11. P1_Q8_AUTRE

Si "autre" précisez

La réponse est obligatoire.

La question n'est pertinente que si P1_Q8 parmi "Autre".

12. P2_Q1

1) Avez-vous dans votre centre un protocole écrit de prescription de corticothérapie précoce à visée préventive respiratoire.

Oui ; Non ; Ne sais pas / Ne se prononce pas

La réponse est obligatoire.

13. P2_Q2

2) Utilisez-vous dans votre centre de l'HYDROCORTISONE prophylactique pour prévenir la survenue de DBP ?

Oui en systématique (sauf contre-indications) ; Oui de manière sélective (administration ciblée sur les patients les plus à risque) ; Cela dépend des praticiens ; Non ; Ne sais pas / Ne se prononce pas

La réponse est obligatoire.

14. P2_Q3

3) Dans votre centre, utilisez-vous une autre stratégie de corticothérapie précoce préventive (durant la 1ère semaine de vie)?

Corticothérapie inhalée ; Autre corticoïdes ; Non ; Ne sais pas / Ne se prononce pas

La réponse est obligatoire.

15. P2_Q3_AUTRE

Si "autre corticoïdes" précisez

La réponse est obligatoire.

La question n'est pertinente que si P2_Q3 parmi "Autre corticoïdes".

16. P2_Q3BIS

Pourriez-vous indiquer les raisons qui vous conduisent à ne pas prescrire de corticothérapie préventive précoce?

La réponse est obligatoire.

La question n'est pertinente que si P2_Q2 parmi "Non" Et P2_Q3 parmi "Non".

17. P2_Q4

4) Dans votre centre, utilisez- vous un algorithme de prédiction de la DBP?

Oui ; Parfois ; Non ; Ne sais pas / Ne se prononce pas

La réponse est obligatoire.

18. P2_Q4BIS

Si oui, précisez lequel

La réponse est obligatoire.

La question n'est pertinente que si P2_Q4 parmi "Oui".

19. P2_Q5

5) Dans votre centre de façon générale, les parents sont-ils informés de l'administration d'HYDROCORTISONE à visée préventive, des bénéfices attendus et des effets indésirables potentiels?

Toujours ; Souvent ; Parfois ; Jamais ; Ne sais pas / Ne se prononce pas

La réponse est obligatoire.

20. P2_Q6

6) En règle générale dans votre centre, le consentement des parents est-il demandé avant d'initier un traitement par corticothérapie préventive précoce?

Toujours ; Souvent ; Parfois ; Jamais ; Nous n'utilisons pas de corticothérapie préventive précoce ; Ne sais pas / Ne se prononce pas

La réponse est obligatoire.

21. P2_Q7

7) Si vous utilisez de l'HYDROCORTISONE précoce, la population cible dans votre centre est la plupart du temps:

Identique ou presque à celle décrite dans l'étude PREMILOC ; Modifiée par rapport à celle de l'étude PREMILOC ; Nous n'utilisons jamais ou presque l'HYDROCORTISONE prophylactique ; Ne sais pas / Ne se prononce pas

La réponse est obligatoire.

22. P2_Q8

8) Pouvez-vous précisez alors vos indications ?

La réponse est obligatoire.

La question n'est pertinente que si P2_Q7 parmi "Modifiée par rapport à celle de l'étude PREMILOC".

23. P2_Q9

9) Si vous utilisez de l'HYDROCORTISONE précoce, l'initiation, la dose et la durée du traitement dans votre centre sont la plupart du temps:

Identiques ou presque à celles décrites dans l'étude PREMILOC ; Différentes en certains points par rapports à celles décrites dans l'étude PREMILOC ; Nous n'utilisons jamais ou presque d'HYDROCORTISONE prophylactique ; Ne sais pas / Ne se prononce pas

La réponse est obligatoire.

24. P2_Q10

10) Pouvez-vous précisez ?

La réponse est obligatoire.

La question n'est pertinente que si P2_Q9 parmi "Différentes en certains points par rapports à celles décrites dans l'étude PREMILOC".

25. P2_Q11

11) l'utilisation d'HYDROCORTISONE vous semble-t-elle avoir eu un impact, en diminuant le recours à l'ibuprofène dans votre centre?

Aucun impact ; Impact minime ; Impact modéré ; Impact important ; Non concerné ; Ne sais pas / Ne se prononce pas

La réponse est obligatoire.

26. P2_Q12

12) Dans votre centre, quels peuvent être les freins à l'utilisation de l'HYDROCORTISONE précoce pour prévenir la DBP? (plusieurs choix possibles)

Aucun frein ; Le risque de perforation digestive ; Le risque infectieux ; Le risque d'Hypertension artérielle ; Le risque d'hyperglycémie ; Le risque de cardiomyopathie hypertrophique ; Le risque potentiel neurodéveloppemental ; Le manque de recul sur cet usage ; Le fait que les résultats de PREMILOC n'aient pas été confirmés par d'autres études ; L'incidence de DBP faible dans votre unité ; Le taux de mortalité faible dans votre unité ; Autre ; Ne sais pas / Ne se prononce pas

La réponse est obligatoire.

Cochez au maximum 6 cases.

27. P2_Q12_AUTRE

Si "autre" précisez

La réponse est obligatoire.

La question n'est pertinente que si P2_Q12 parmi "Autre".

28. P2_Q13

13) Dans votre centre, quelles peuvent être les motivations à l'utilisation d'HYDROCORTISONE précoce pour prévenir la DBP ? (plusieurs choix possibles)

Eviter une ventilation invasive ; Permettre à l'enfant d'être globalement plus stable pensant la période de traitement ; Avoir un effet concomitant positif sur la tension artérielle ; Faciliter l'extubation ; Faciliter la fermeture spontanée du canal artériel ; Réduire le risque de dysnatrémie ; Diminuer la FiO2 ; Supplémenter une insuffisance surrénale physiologique ; L'augmentation de la survie sans dysplasie bronchopulmonaire ; Autre ; Ne sais pas / Ne se prononce pas

La réponse est obligatoire.

Cochez au maximum 2 cases.

29. P2_Q13_AUTRE

Si "autre" précisez

La réponse est obligatoire.

La question n'est pertinente que si P2_Q13 parmi "Autre".

30. P2_Q14

14) L'utilisation d'HYDROCORTISONE vous semble-t-elle avoir eu des effets (réels ou subjectifs) sur : (plusieurs choix possibles)

La stabilité globale de l'enfant ; L'augmentation des perforations intestinales ; La diminution du recours à un traitement médical ou chirurgical du PDA ; La diminution du recours à la ventilation invasive ; La correction de troubles hydroélectrolytiques ; L'augmentation des hyperglycémies et du recours à l'insuline ; Nous n'utilisons pas l'hydrocortisone ; Autre ; Ne sais pas / Ne se prononce pas

La réponse est obligatoire.

Cochez au maximum 2 cases.

31. P2_Q14_AUTRE

Si "autre" précisez

La réponse est obligatoire.

La question n'est pertinente que si P2_Q14 parmi "Autre".

32. P2_Q15

15) Dans votre centre, utilisez-vous l'HYDROCORTISONE chez l'enfant prématuré pour d'autres indications?

Oui ; Non ; Ne se prononce pas

La réponse est obligatoire.

33. P2_Q15BIS

Si oui : (plusieurs choix possibles)

Indication hémodynamique ; Indication de substitution de la fonction surrénalienne ; Autre ; Ne sais pas / Ne se prononce pas

La réponse est obligatoire.

Vous pouvez cocher plusieurs cases.

La question n'est pertinente que si P2_Q15 parmi "Oui".

34. P2_Q15BIS_AUTRE

Si "autre" précisez

La réponse est obligatoire.

La question n'est pertinente que si P2_Q15BIS parmi "Autre".

35. P2_Q16

16) Dans votre centre la plupart du temps, un dosage de cortisol est-il réalisé avant l'instauration d'un traitement par HYDROCORTISONE?

Oui ; Parfois ; Non ; Ne sais pas / Ne se prononce pas

La réponse est obligatoire.

36. P3_Q1

1) Dans votre centre avez-vous un protocole écrit d'utilisation de corticothérapie à visée respiratoire tardive?

Oui ; Non ; Ne sais pas / Ne se prononce pas

La réponse est obligatoire.

37. P3_Q2

2) Dans votre centre de façon générale, les parents sont-ils informés de l'administration d'un traitement par corticothérapie tardive, des bénéfices attendus et des effets indésirables potentiels?

Toujours ; Souvent ; Parfois ; Jamais ; Nous n'utilisons pas de corticothérapie postnatale ; Ne sais pas / Ne se prononce pas

La réponse est obligatoire.

38. P3_Q3

3) En règle générale dans votre centre, le consentement des parents est-il demandé avant d'initier un traitement par corticothérapie tardive?

Toujours ; Souvent ; Parfois ; Jamais ; Nous n'utilisons pas de corticothérapie postnatale ; Ne sais pas / Ne se prononce pas

La réponse est obligatoire.

39. P3_Q4

4) Dans votre centre en règle générale, quelle(s) molécule(s) est/sont utilisées, quand vous avez recours à de la corticothérapie à visée respiratoire curative, ou préventive tardive? (plusieurs réponses possibles)

Betaméthasone ; Dexamethasone ; Hydrocortisone ; Fluticasone ; Budesonide ; Nous n’utilisons pas de corticothérapie postnatale ; Autre ; Ne sais pas / Ne se prononce pas

La réponse est obligatoire.

Vous pouvez cocher plusieurs cases.

40. P3_Q4_AUTRE

Si "autre" précisez

La réponse est obligatoire.

La question n'est pertinente que si P3_Q4 parmi "Autre".

41. P3_Q5

5) Dans votre centre en règle générale, quelle voie utilisez-vous pour traiter par corticothérapie postnatale tardive (plusieurs réponses possibles)

Per os ; IV même si la voie Per Os est possible ; IV seulement si la voie per os n'est pas possible ; Inhalée ; Nous n’utilisons pas de corticothérapie postnatale ; Ne sais pas / Ne se prononce pas

La réponse est obligatoire.

Vous pouvez cocher plusieurs cases.

42. P3_Q6

La réponse est obligatoire. La réponse doit être comprise entre 8 et 30.

La question n'est pertinente que si P3_Q6BIS parmi "Non-réponse".

43. P3_Q6BIS

Ne sais pas / Ne se prononce pas

44. P3_Q7

7) Dans votre centre en règle générale, vous utilisez la corticothérapie tardive chez des enfants: (plusieurs réponses possibles)

En ventilation invasive ; En DuoPap ou autre ventilation non invasive à 2 niveaux de pression ; En CPAP simple ; En lunettes OHD ; Nous n’utilisons pas de corticothérapie postnatale ; Autre ; Ne sais pas / Ne se prononce pas

La réponse est obligatoire.

Vous pouvez cocher plusieurs cases.

45. P3_Q7_AUTRE

Si "autre" précisez

La réponse est obligatoire.

La question n'est pertinente que si P3_Q7 parmi "Autre".

46. P3_Q8

8) Dans votre centre, quelles peuvent être les motivations à recourir à une corticothérapie tardive chez des enfants symptomatiques sur le plan respiratoire? (plusieurs choix possibles)

Faciliter l’extubation ; Éviter une réintubation ; Sevrer de la ventilation non invasive ; Diminuer la FiO2 ;

Réduire la durée totale d’oxygénothérapie ; Prévenir la survenue de DBP à 36SA ; Eviter un retour à domicile sous oxygène ; Améliorer le pronostic respiratoire à long terme ; Améliorer le pronostic global à long terme ; Réduire la mortalité ; Nous n’utilisons pas de corticothérapie postnatale ; Autre ; Ne sais pas / Ne se prononce pas

La réponse est obligatoire.

Vous pouvez cocher plusieurs cases.

47. P3_Q8_AUTRE

Si "autre" précisez

La réponse est obligatoire.

La question n'est pertinente que si P3_Q8 parmi "Autre".

48. P3_Q9

9) Dans votre centre en règle générale, à partir de quel taux de FiO2 en VENTILATION INVASIVE envisagez-vous une corticothérapie tardive?

30% ; 40% ; 50% ; 60% ; Nous n’utilisons pas de corticothérapie postnatale ; Autre ; Ne sais pas / Ne se prononce pas

La réponse est obligatoire.

49. P3_Q9_AUTRE

Si "autre" précisez

La réponse est obligatoire.

La question n'est pertinente que si P3_Q9 parmi "Autre".

50. P3_Q10

10) Dans votre centre en règle générale, à partir de quel taux de FiO2 en VENTILATION NON INVASIVE envisagez-vous une corticothérapie tardive ?

30% ; 40% ; 50% ; 60% ; Autre ; Nous n’utilisons pas de corticothérapie postnatale ; Ne sais pas / Ne se prononce pas

La réponse est obligatoire.

51. P3_Q10_AUTRE

Si "autre" précisez

La réponse est obligatoire.

La question n'est pertinente que si P3_Q10 parmi "Autre".

52. P3_Q11

11) De façon générale dans votre centre, vous arrive-t-il de faire des cures multiples de corticothérapie tardive ?

Jamais ; Parfois ; Souvent ; Toujours ; Ne sais pas / Ne se prononce pas

La réponse est obligatoire.

53. P3_Q12

12) Dans votre centre, quels peuvent être les freins à recourir à une corticothérapie tardive chez des enfants symptomatiques sur le plan respiratoire ? (plusieurs choix possibles)

Aucun frein ; Le risque de perforation digestive ; Le risque neurodéveloppemental ; Le risque infectieux ; Le risque d’Hypertension artérielle ; Le risque d’hyperglycémie ; Le risque de cardiomyopathie hypertrophique ; Le risque ophtalmologique ; Le risque médico-légal ; Autre ; Nous n’utilisons pas de

corticothérapie postnatale ; Ne sais pas / Ne se prononce pas

La réponse est obligatoire.

Vous pouvez cocher plusieurs cases.

54. P3_Q12_AUTRE

Si "autre" précisez

La réponse est obligatoire.

La question n'est pertinente que si P3_Q12 parmi "Autre".

55. P4_Q1

1) A titre individuel, connaissez-vous les dernières recommandations françaises d'utilisation de la corticothérapie postnatale chez l'enfant né prématuré

Oui ; Non ; Partiellement ; Ne sais pas / Ne se prononce pas

La réponse est obligatoire.

56. P4_Q2

2) En règle générale dans votre centre, ces recommandations sont appliquées

Toujours ; Souvent ; Parfois ; Jamais ; Ne sais pas / Ne se prononce pas

La réponse est obligatoire.

57. P4_Q3

3) Pensez-vous qu'il serait utile de mettre à jour les recommandations d'utilisation de la corticothérapie postnatale chez l'enfant né prématuré ?

Oui ; Non ; Ne sais pas / Ne se prononce pas

La réponse est obligatoire.

58. P4_Q4

4) D’après vous, quelles questions devraient traiter ces nouvelles recommandations? (plusieurs réponses possibles)

Le type de molécule à utiliser ; Le timing d‘utilisation ; La dose ; La durée ; La population cible pour une corticothérapie préventive systématique ; La population cible pour une corticothérapie tardive curative ; La surveillance clinique et paraclinique lors d’une cure de corticothérapie ; Le bilan à réaliser avant une cure de corticothérapie ; Autre ; Ne sais pas / Ne se prononce pas

La réponse est obligatoire.

Vous pouvez cocher plusieurs cases.

59. P4_Q4_AUTRE

Si "autre" précisez

La réponse est obligatoire.

La question n'est pertinente que si P4_Q4 parmi "Autre".

60. P4_Q5

Dans votre centre, quelles sont les interrogations et débats que vous pouvez rencontrer au quotidien dans vos équipe concernant la corticothérapie postnatale chez les enfants nés extrêmes et grands prématurés ?

La réponse est obligatoire.

61. P4_Q6

6) Seriez-vous intéressé pour participer à un groupe de travail sur la rédaction de ces nouvelles

recommandations ?

Oui ; Non ; Ne sais pas / Ne se prononce pas

La réponse est obligatoire.

Variables de publication

62. CLE

La réponse est automatique (clé primaire).

63. DATE_SAISIE

La réponse est automatique. "jj/mm/aaaa hh:mm:ss".

64. DATE_ENREG

La réponse est automatique. "jj/mm/aaaa hh:mm:ss".

65. DATE_MODIF

La réponse est automatique. "jj/mm/aaaa hh:mm:ss".

66. TEMPS_SAISIE

La réponse est automatique.

67. ORIGINE_SAISIE

68. LANG_SAISIE

La réponse est automatique.

69. APPAREIL_SAISIE

PC ; Tablette ; Smartphone

La réponse est automatique.

70. PROGRESSION

En cours ; Terminé ; QuotaFull ; ScreenOut

La réponse est automatique.
